# 4-Chloro-3-methyl­phenyl quinoline-2-carboxyl­ate

**DOI:** 10.1107/S1600536813032017

**Published:** 2013-11-30

**Authors:** E. Fazal, Manpreet Kaur, B. S. Sudha, S. Nagarajan, Jerry P. Jasinski

**Affiliations:** a Department of Chemistry, Yuvaraja’s College, Mysore 570 005, India; b Department of Studies in Chemistry, University of Mysore, Manasagangotri, Mysore 570 006, India; c P.P.S.F.T Department, Central Food Technology Research institute, Mysore 570 005, India; d Department of Chemistry, Keene State College, 229 Main Street, Keene, NH 03435-2001, USA

## Abstract

In the title compound, C_17_H_12_ClNO_2_, the dihedral angle between the mean planes of the quinoline ring system and the benzene ring is 68.7 (7)°. The mean plane of the carboxyl­ate group is twisted from the latter planes by 14.0 (1) and 80.2 (4)°, respectively. In the crystal, weak C—H⋯O inter­actions are observed, forming chains along [001]. In addition, π–π stacking inter­actions [centroid–centroid distances = 3.8343 (13) and 3.7372 (13)Å] occur. No classical hydrogen bonds were observed.

## Related literature

For heterocycles in natural products, see: Morimoto *et al.* (1991 ▶); Michael (1997 ▶). For heterocycles in fragrances and dyes, see: Padwa *et al.* (1999 ▶). For heterocycles in biologically active compounds, see: Markees *et al.* (1970 ▶); Campbell *et al.* (1988 ▶). For the use of quinoline alkaloids as efficient drugs for the treatment of malaria, see: Robert & Meunier, (1998 ▶). For quinoline as a privileged scaffold in cancer drug discovery, see: Solomon & Lee (2011 ▶). For related structures, see: Fazal *et al.* (2012 ▶); Butcher *et al.* (2007 ▶); Jing & Qin (2008 ▶); Jasinski *et al.* (2010 ▶).
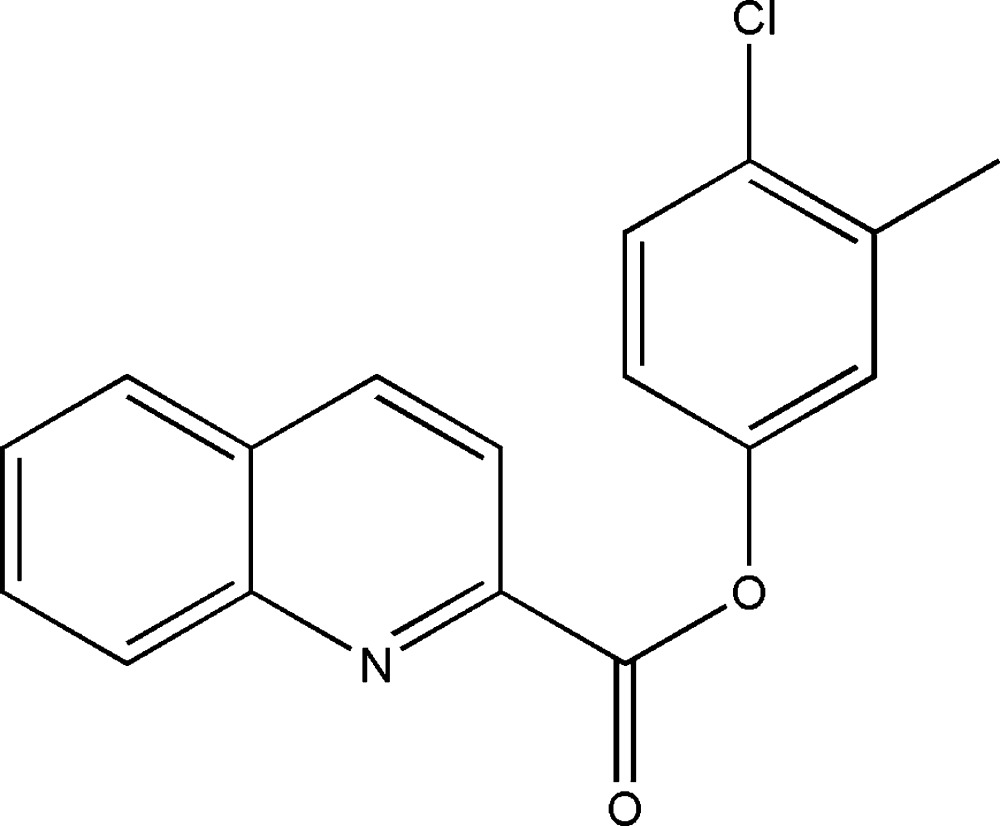


## Experimental

### 

#### Crystal data

C_17_H_12_ClNO_2_
*M* *_r_* = 297.73
Orthorhombic, 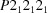

*a* = 7.75379 (16) Å
*b* = 11.9658 (3) Å
*c* = 14.9005 (3) Å
*V* = 1382.48 (5) Å^3^
*Z* = 4
Cu *K*α radiation
μ = 2.48 mm^−1^
*T* = 173 K
0.32 × 0.24 × 0.20 mm

#### Data collection

Agilent Xcalibur (Eos, Gemini) diffractometer
Absorption correction: multi-scan (*CrysAlis PRO* and *CrysAlis RED*; Agilent, 2012 ▶) *T*_min_ = 0.530, *T*_max_ = 1.000
8419 measured reflections
2703 independent reflections
2636 reflections with *I* > 2σ(*I*)
*R*_int_ = 0.030

#### Refinement

*R*[*F* ^2^ > 2σ(*F* ^2^)] = 0.032
*wR*(*F* ^2^) = 0.085
*S* = 1.05
2703 reflections
192 parameters
H-atom parameters constrained
Δρ_max_ = 0.20 e Å^−3^
Δρ_min_ = −0.19 e Å^−3^
Absolute structure: Flack parameter determined using 1081 quotients [(*I* ^+^)−(*I* ^−^)]/[(*I* ^+^)+(*I* ^−^)] (Parsons *et al.*, 2013 ▶)
Absolute structure parameter: −0.009 (10)

### 

Data collection: *CrysAlis PRO* (Agilent, 2012 ▶); cell refinement: *CrysAlis PRO*; data reduction: *CrysAlis RED* (Agilent, 2012 ▶); program(s) used to solve structure: *SUPERFLIP* (Palatinus & Chapuis, 2007 ▶); program(s) used to refine structure: *SHELXL2012* (Sheldrick, 2008 ▶); molecular graphics: *OLEX2* (Dolomanov *et al.*, 2009 ▶); software used to prepare material for publication: *OLEX2*.

## Supplementary Material

Crystal structure: contains datablock(s) I. DOI: 10.1107/S1600536813032017/bt6947sup1.cif

Structure factors: contains datablock(s) I. DOI: 10.1107/S1600536813032017/bt6947Isup2.hkl

Supplementary material file. DOI: 10.1107/S1600536813032017/bt6947Isup3.cml

Additional supplementary materials:  crystallographic information; 3D view; checkCIF report

## Figures and Tables

**Table 1:** Hydrogen-bond geometry (Å, °)

*D*—H⋯*A*	*D*—H	H⋯*A*	*D*⋯*A*	*D*—H⋯*A*
C8—H8⋯O1^i^	0.93	2.57	3.317 (3)	138

Symmetry code: (i) 
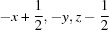
.

**Fig. 1.**
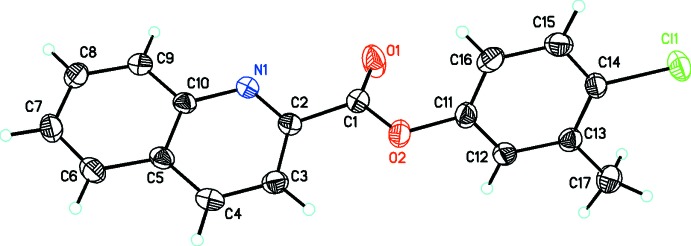
ORTEP drawing of the title compound showing the labeling scheme with 50% probability displacement ellipsoids.

**Fig. 2.**
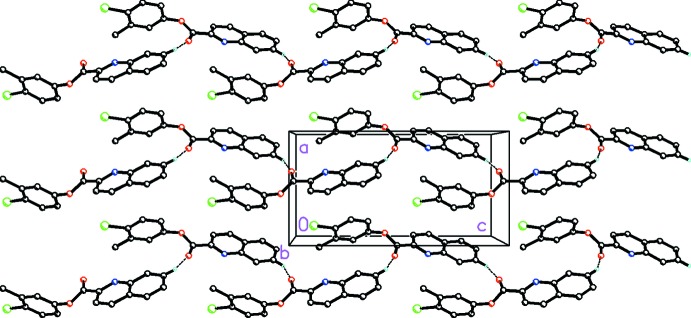
Molecular packing of the title compound viewed along the b axis. Dashed lines indicate weak C8—H8···O1 intermolecular interactions making chains along [0 0 1] and influence the crystal packing. The remaining H atoms have been removed for clarity.

## supplementary crystallographic information

### 1. Comment

Quinoline-2 carboxylic acid derivatives are a class of important materials as anti-tuberculosis agents, as fluorescent reagents, hydrophobic field-detection reagents, visualisation reagents, fluorescent labelled peptide probes and as antihyperglycemics. Quinoline derivatives represent a major class of heterocycles and are found in natural products (Morimoto *et al.*, 1991; Michael, 1997), numerous commercial products, including fragrances, dyes (Padwa *et al.*, 1999) and biologically active compounds (Markees *et al.*, 1970; Campbell *et al.*, 1988). Quinoline alkaloids such as quinine, chloroquin, mefloquine and amodiaquine are used as efficient drugs for the treatment of malaria (Robert & Meunier, 1998). Quinoline as a privileged scaffold in cancer drug discovery is published (Solomon & Lee, 2011). The crystal structures of 4-methylphenyl quinoline-2-carboxylate (Fazal *et al.*, 2012), 1-(quinolin-2-yl)ethanone (Butcher *et al.*, 2007) and methyl quinoline-2-carboxylate (Jing & Qin, 2008) and the synthesis, crystal structures and theoretical studies of four Schiff bases derived from 4-hydrazinyl-8-(trifluoromethyl) quinoline (Jasinski *et al.*, 2010) have been reported. In view of the importance of quinolines, this paper reports the crystal structure of the title compound, C_17_H_12_ClNO_2_.

In the title compound, the dihedral angle between the mean planes of the quinoline ring and the phenyl ring is 68.7 (7)° (Fig. 1). The mean plane of the carboxylate group is twisted from the mean planes of the quinoline ring and phenyl ring by 14.0 (1)° and 80.2 (4)°, respectively. In the crystal, weak C8—H8···O1 intermolecular interactions are observed making chains along [0 0 1] (Fig. 2). In addition, π–π stacking interactions further stabilize the crystal packing with centroid-centroid distances of 3.8343 (13)Å (Cg1–Cg3^i^) and 3.7372 (13)Å (Cg2–Cg3^i^) (where Cg1 = N1/C2/C3/C4/C5/C10; Cg2 = C5-C10; Cg3 = C11-C16; symmetry operator (i) -0.5+*x*, 0.5-*y*, 1-*z*). No classical hydrogen bonds were observed.

### 2. Experimental

The title compound was prepared by the following procedure: To a mixture of 1.73 g (10 mmole) of quinaldic acid and 1.42 g (10 mmole) of 4-chloro-3-methylphenol in a round-bottomed flask fitted with a reflex condenser with a drying tube is added 0.75 g (5 mmole) of phosphorous oxychloride. The mixture is heated with occasional swirling, and temperature is maintained at 353-363 K. At the end of eight hours the reaction mixture is poured in to a solution of 2 g of sodium bicarbonate in 25 mL of water. The precipitated ester is collected on a filter and washed with water. The yield of crude, air dried 4-chloro-3-methyl phenyl quinoline-2-carboxylate is 1.89 to 2.05 g (60-70%). X-ray quality crystal was obtained by recrystallization from absolute ethanol (M.P.: 383-385 K).

### 3. Refinement

All H atoms were visible in a difference map, but placed in their calculated positions and then refined using the riding model with Atom—H lengths of 0.93Å (CH) or 0.96Å (CH_3_). Isotropic displacement parameters for these atoms were set to 1.2 (CH) or 1.5 (CH_3_) times *U*_eq_ of the parent atom. Idealised Me refined as rotating group.

### Crystal data

**Table:**

C_17_H_12_ClNO_2_	*D*_x_ = 1.430 Mg m^−^^3^
*M**_r_* = 297.73	Cu *K*α radiation, λ = 1.54184 Å
Orthorhombic, *P*2_1_2_1_2_1_	Cell parameters from 4795 reflections
*a* = 7.75379 (16) Å	θ = 4.7–72.2°
*b* = 11.9658 (3) Å	µ = 2.48 mm^−^^1^
*c* = 14.9005 (3) Å	*T* = 173 K
*V* = 1382.48 (5) Å^3^	Irregular, colourless
*Z* = 4	0.32 × 0.24 × 0.20 mm
*F*(000) = 616	

### Data collection

**Table:**

Agilent Xcalibur (Eos, Gemini) diffractometer	2703 independent reflections
Radiation source: Enhance (Cu) X-ray Source	2636 reflections with *I* > 2σ(*I*)
Detector resolution: 16.0416 pixels mm^-1^	*R*_int_ = 0.030
ω scans	θ_max_ = 72.3°, θ_min_ = 4.7°
Absorption correction: multi-scan (*CrysAlis PRO* and *CrysAlis RED*; Agilent, 2012)	*h* = −9→4
*T*_min_ = 0.530, *T*_max_ = 1.000	*k* = −14→14
8419 measured reflections	*l* = −17→18

### Refinement

**Table:**

Refinement on *F*^2^	H-atom parameters constrained
Least-squares matrix: full	*w* = 1/[σ^2^(*F*_o_^2^) + (0.0574*P*)^2^ + 0.1134*P*] where *P* = (*F*_o_^2^ + 2*F*_c_^2^)/3
*R*[*F*^2^ > 2σ(*F*^2^)] = 0.032	(Δ/σ)_max_ < 0.001
*wR*(*F*^2^) = 0.085	Δρ_max_ = 0.20 e Å^−^^3^
*S* = 1.05	Δρ_min_ = −0.19 e Å^−^^3^
2703 reflections	Extinction correction: *SHELXL2012* (Sheldrick, 2008), Fc^*^=kFc[1+0.001xFc^2^λ^3^/sin(2θ)]^-1/4^
192 parameters	Extinction coefficient: 0.0036 (6)
0 restraints	Absolute structure: Flack parameter determined using 1081 quotients [(*I*^+^)-(*I*^-^)]/[(*I*^+^)+(*I*^-^)] (Parsons *et al.*, 2013)
Primary atom site location: structure-invariant direct methods	Absolute structure parameter: −0.009 (10)
Hydrogen site location: inferred from neighbouring sites	

### Special details

**Table:**

Experimental. ^1^HNMR(500 MHz,DMSO) δ 8.66 (1H,d, J= 8.51Hz), 8.27(1H,d, J= 8.5Hz),8.24(1H,d, J= 8.43 Hz), 8.15(1H,d, J= 8.2 Hz),7.93(1H,dt, J1= 8.07Hz, J2=6.73, J3=1.06Hz), 7.8(1H,t, J= 7.55Hz), 7.54(1H,d, J= 8.6Hz), 7.41(1H,d, J= 2.4Hz), 7.26(1H,dd, J1= 8.6Hz, J2=2.57 Hz), 3.3-3.4(1H,m),2.38(3H,s).
Geometry. All esds (except the esd in the dihedral angle between two l.s. planes) are estimated using the full covariance matrix. The cell esds are taken into account individually in the estimation of esds in distances, angles and torsion angles; correlations between esds in cell parameters are only used when they are defined by crystal symmetry. An approximate (isotropic) treatment of cell esds is used for estimating esds involving l.s. planes.

### Fractional atomic coordinates and isotropic or equivalent isotropic displacement parameters (Å^2^)

**Table:**

	*x*	*y*	*z*	*U*_iso_*/*U*_eq_	
Cl1	0.64546 (8)	−0.08068 (5)	0.89548 (4)	0.03791 (19)	
O1	0.3575 (3)	0.03286 (15)	0.50511 (12)	0.0448 (5)	
O2	0.5593 (2)	0.15377 (14)	0.55202 (10)	0.0319 (4)	
N1	0.3627 (2)	0.13829 (14)	0.33957 (12)	0.0241 (4)	
C1	0.4441 (3)	0.11335 (17)	0.49220 (14)	0.0262 (5)	
C2	0.4431 (3)	0.18389 (18)	0.40823 (14)	0.0244 (4)	
C3	0.5281 (3)	0.28841 (18)	0.40610 (15)	0.0271 (5)	
H3	0.5787	0.3179	0.4575	0.032*	
C4	0.5337 (3)	0.34478 (19)	0.32646 (16)	0.0284 (5)	
H4	0.5880	0.4140	0.3230	0.034*	
C5	0.4568 (3)	0.29779 (17)	0.24938 (15)	0.0244 (4)	
C6	0.4651 (3)	0.34833 (19)	0.16312 (16)	0.0305 (5)	
H6	0.5219	0.4162	0.1557	0.037*	
C7	0.3899 (3)	0.2973 (2)	0.09138 (16)	0.0343 (5)	
H7	0.3987	0.3296	0.0348	0.041*	
C8	0.2985 (3)	0.1955 (2)	0.10181 (16)	0.0324 (5)	
H8	0.2467	0.1623	0.0523	0.039*	
C9	0.2861 (3)	0.14592 (19)	0.18406 (15)	0.0276 (5)	
H9	0.2233	0.0802	0.1905	0.033*	
C10	0.3685 (3)	0.19422 (18)	0.25977 (14)	0.0231 (4)	
C11	0.5754 (3)	0.09412 (19)	0.63323 (14)	0.0270 (5)	
C12	0.4958 (3)	0.13691 (19)	0.70842 (15)	0.0259 (4)	
H12	0.4295	0.2014	0.7039	0.031*	
C13	0.5141 (3)	0.08374 (19)	0.79167 (14)	0.0255 (4)	
C14	0.6158 (3)	−0.01188 (18)	0.79341 (15)	0.0265 (5)	
C15	0.6979 (3)	−0.05421 (19)	0.71832 (16)	0.0305 (5)	
H15	0.7661	−0.1179	0.7226	0.037*	
C16	0.6771 (3)	−0.00042 (19)	0.63626 (15)	0.0305 (5)	
H16	0.7305	−0.0275	0.5848	0.037*	
C17	0.4262 (3)	0.1281 (2)	0.87402 (15)	0.0340 (5)	
H17A	0.3664	0.1960	0.8594	0.051*	
H17B	0.5107	0.1430	0.9196	0.051*	
H17C	0.3452	0.0738	0.8957	0.051*	

### Atomic displacement parameters (Å^2^)

**Table:**

	*U*^11^	*U*^22^	*U*^33^	*U*^12^	*U*^13^	*U*^23^
Cl1	0.0486 (3)	0.0340 (3)	0.0312 (3)	−0.0002 (3)	−0.0084 (2)	0.0109 (2)
O1	0.0585 (11)	0.0405 (10)	0.0356 (9)	−0.0239 (9)	−0.0172 (9)	0.0143 (8)
O2	0.0431 (9)	0.0316 (8)	0.0209 (7)	−0.0097 (7)	−0.0046 (7)	0.0033 (6)
N1	0.0279 (8)	0.0201 (8)	0.0241 (8)	−0.0002 (8)	−0.0002 (7)	0.0010 (7)
C1	0.0301 (10)	0.0250 (11)	0.0235 (10)	−0.0002 (9)	−0.0018 (9)	−0.0006 (8)
C2	0.0267 (10)	0.0213 (9)	0.0251 (10)	0.0028 (8)	0.0001 (8)	0.0000 (8)
C3	0.0298 (11)	0.0229 (10)	0.0285 (11)	−0.0003 (8)	−0.0029 (9)	−0.0029 (9)
C4	0.0299 (11)	0.0193 (10)	0.0360 (11)	−0.0009 (8)	0.0009 (9)	0.0000 (9)
C5	0.0256 (10)	0.0194 (9)	0.0282 (10)	0.0040 (8)	0.0043 (8)	0.0017 (8)
C6	0.0345 (12)	0.0238 (11)	0.0331 (11)	0.0032 (9)	0.0064 (9)	0.0064 (9)
C7	0.0447 (14)	0.0325 (12)	0.0256 (11)	0.0071 (10)	0.0060 (10)	0.0066 (9)
C8	0.0407 (12)	0.0319 (12)	0.0246 (10)	0.0060 (9)	−0.0007 (9)	−0.0031 (9)
C9	0.0325 (10)	0.0221 (10)	0.0282 (11)	0.0023 (9)	−0.0002 (9)	−0.0011 (9)
C10	0.0247 (10)	0.0196 (9)	0.0250 (10)	0.0040 (8)	0.0019 (8)	0.0004 (8)
C11	0.0320 (10)	0.0274 (11)	0.0216 (10)	−0.0082 (9)	−0.0049 (8)	0.0025 (8)
C12	0.0277 (10)	0.0230 (10)	0.0269 (10)	−0.0016 (9)	−0.0038 (8)	0.0000 (9)
C13	0.0264 (10)	0.0254 (10)	0.0247 (10)	−0.0047 (9)	−0.0021 (8)	−0.0009 (9)
C14	0.0310 (11)	0.0249 (10)	0.0236 (9)	−0.0045 (9)	−0.0060 (8)	0.0041 (8)
C15	0.0338 (12)	0.0222 (11)	0.0354 (12)	0.0004 (9)	−0.0032 (9)	−0.0025 (9)
C16	0.0352 (12)	0.0299 (11)	0.0263 (10)	−0.0024 (9)	0.0007 (9)	−0.0070 (9)
C17	0.0378 (12)	0.0375 (12)	0.0268 (11)	0.0003 (10)	0.0050 (9)	−0.0004 (9)

### Geometric parameters (Å, º)

**Table:**

Cl1—C14	1.745 (2)	C8—H8	0.9300
O1—C1	1.190 (3)	C8—C9	1.365 (3)
O2—C1	1.352 (3)	C9—H9	0.9300
O2—C11	1.410 (2)	C9—C10	1.420 (3)
N1—C2	1.317 (3)	C11—C12	1.378 (3)
N1—C10	1.365 (3)	C11—C16	1.380 (3)
C1—C2	1.509 (3)	C12—H12	0.9300
C2—C3	1.414 (3)	C12—C13	1.401 (3)
C3—H3	0.9300	C13—C14	1.390 (3)
C3—C4	1.366 (3)	C13—C17	1.501 (3)
C4—H4	0.9300	C14—C15	1.383 (3)
C4—C5	1.411 (3)	C15—H15	0.9300
C5—C6	1.422 (3)	C15—C16	1.391 (3)
C5—C10	1.424 (3)	C16—H16	0.9300
C6—H6	0.9300	C17—H17A	0.9600
C6—C7	1.362 (4)	C17—H17B	0.9600
C7—H7	0.9300	C17—H17C	0.9600
C7—C8	1.418 (4)		
			
C1—O2—C11	116.31 (17)	C10—C9—H9	119.8
C2—N1—C10	117.25 (18)	N1—C10—C5	122.49 (19)
O1—C1—O2	123.8 (2)	N1—C10—C9	118.53 (19)
O1—C1—C2	125.7 (2)	C9—C10—C5	119.0 (2)
O2—C1—C2	110.48 (18)	C12—C11—O2	118.0 (2)
N1—C2—C1	114.51 (18)	C12—C11—C16	122.3 (2)
N1—C2—C3	124.7 (2)	C16—C11—O2	119.6 (2)
C3—C2—C1	120.73 (19)	C11—C12—H12	119.8
C2—C3—H3	120.9	C11—C12—C13	120.4 (2)
C4—C3—C2	118.1 (2)	C13—C12—H12	119.8
C4—C3—H3	120.9	C12—C13—C17	121.1 (2)
C3—C4—H4	120.1	C14—C13—C12	116.6 (2)
C3—C4—C5	119.8 (2)	C14—C13—C17	122.3 (2)
C5—C4—H4	120.1	C13—C14—Cl1	118.67 (17)
C4—C5—C6	123.2 (2)	C15—C14—Cl1	118.13 (17)
C4—C5—C10	117.5 (2)	C15—C14—C13	123.2 (2)
C6—C5—C10	119.4 (2)	C14—C15—H15	120.4
C5—C6—H6	120.0	C14—C15—C16	119.2 (2)
C7—C6—C5	119.9 (2)	C16—C15—H15	120.4
C7—C6—H6	120.0	C11—C16—C15	118.3 (2)
C6—C7—H7	119.6	C11—C16—H16	120.9
C6—C7—C8	120.9 (2)	C15—C16—H16	120.9
C8—C7—H7	119.6	C13—C17—H17A	109.5
C7—C8—H8	119.8	C13—C17—H17B	109.5
C9—C8—C7	120.5 (2)	C13—C17—H17C	109.5
C9—C8—H8	119.8	H17A—C17—H17B	109.5
C8—C9—H9	119.8	H17A—C17—H17C	109.5
C8—C9—C10	120.3 (2)	H17B—C17—H17C	109.5
			
Cl1—C14—C15—C16	−179.57 (17)	C6—C5—C10—C9	−2.0 (3)
O1—C1—C2—N1	−13.1 (3)	C6—C7—C8—C9	−0.8 (4)
O1—C1—C2—C3	168.7 (2)	C7—C8—C9—C10	−1.7 (4)
O2—C1—C2—N1	166.49 (19)	C8—C9—C10—N1	−175.7 (2)
O2—C1—C2—C3	−11.7 (3)	C8—C9—C10—C5	3.1 (3)
O2—C11—C12—C13	−177.10 (18)	C10—N1—C2—C1	−175.21 (17)
O2—C11—C16—C15	176.69 (19)	C10—N1—C2—C3	2.9 (3)
N1—C2—C3—C4	−2.7 (3)	C10—C5—C6—C7	−0.4 (3)
C1—O2—C11—C12	−102.0 (2)	C11—O2—C1—O1	0.7 (3)
C1—O2—C11—C16	81.7 (3)	C11—O2—C1—C2	−178.92 (17)
C1—C2—C3—C4	175.29 (19)	C11—C12—C13—C14	0.3 (3)
C2—N1—C10—C5	−0.1 (3)	C11—C12—C13—C17	−179.1 (2)
C2—N1—C10—C9	178.66 (19)	C12—C11—C16—C15	0.6 (3)
C2—C3—C4—C5	−0.4 (3)	C12—C13—C14—Cl1	179.22 (16)
C3—C4—C5—C6	−176.53 (19)	C12—C13—C14—C15	0.6 (3)
C3—C4—C5—C10	2.9 (3)	C13—C14—C15—C16	−0.9 (3)
C4—C5—C6—C7	179.0 (2)	C14—C15—C16—C11	0.3 (3)
C4—C5—C10—N1	−2.8 (3)	C16—C11—C12—C13	−0.9 (3)
C4—C5—C10—C9	178.5 (2)	C17—C13—C14—Cl1	−1.4 (3)
C5—C6—C7—C8	1.9 (4)	C17—C13—C14—C15	180.0 (2)
C6—C5—C10—N1	176.7 (2)		

### Hydrogen-bond geometry (Å, º)

**Table:**

*D*—H···*A*	*D*—H	H···*A*	*D*···*A*	*D*—H···*A*
C8—H8···O1^i^	0.93	2.57	3.317 (3)	138

Symmetry code: (i) −*x*+1/2, −*y*, *z*−1/2.
